# The functional maturity of grafted human pluripotent stem cell derived-islets (hSC-Islets) evaluated by the glycemic set point during blood glucose normalizing process in diabetic mice

**DOI:** 10.1016/j.heliyon.2023.e19972

**Published:** 2023-09-09

**Authors:** Shigeharu G. Yabe, Satsuki Fukuda, Junko Nishida, Fujie Takeda, Hitoshi Okochi

**Affiliations:** Department of Regenerative Medicine, Research Institute, National Center for Global Health and Medicine, 1-21-1 Toyama Shinjuku-ku, Tokyo, 162-8655, Japan

**Keywords:** Human pluripotent stem cells (hPSCs), Pancreatic islets, Diabetes, Glycemic set point, Revascularization, Reinnervation

## Abstract

Human pluripotent stem cell (hPSCs) derived-pancreatic islets (hSC-islets) are good candidates for cell replacement therapy for patients with diabetes as substitutes for deceased donor-derived islets, because they are pluripotent and have infinite proliferation potential. Grafted hSC-islets ameliorate hyperglycemia in diabetic mice; however, several weeks are needed to normalize the hyperglycemia. These data suggest hSC-islets require maturation, but their maturation process *in vivo* is not yet fully understood. In this study, we utilized two kinds of streptozotocin (STZ)-induced diabetes model mice by changing the administration timing in order to examine the time course of maturation of hSC-islets and the effects of hyperglycemia on their maturation. We found no hyperglycemia in immune-compromised mice when hSC-islets had been transplanted under their kidney capsules in advance, and STZ was administered 4 weeks after transplantation. Of note, the blood glucose levels of those mice were stably maintained under 100 mg/dl 10 weeks after transplantation; this is lower than the mouse glycemic set point (120–150 mg/dl), suggesting that hSC-islets control blood glucose levels to the human glycemic set point. We confirmed that gene expression of maturation markers of pancreatic beta cells tended to upregulate during 4 weeks after transplantation. Periodical histological analysis revealed that revascularization was observed as early as 1 week after transplantation, but reinnervation in the grafted hSC-islets was not detected at all, even 15 weeks after transplantation. In conclusion, our hSC-islets need at least 4 weeks to mature, and the human glycemic set point is a good index for evaluating ultimate maturity for hSC-islets *in vivo*.

## 1 Introduction

In collaboration with glucagon secreting-pancreatic alpha cells and somatostatin-secreting delta cells, pancreatic beta cells play a central role in regulating serum glucose levels within a normal range by adjusting the amount of insulin secretion in response to glucose levels [[1], [2], [3], [4]]. Currently, transplantation of pancreatic islets is useful as therapy for diabetes [[5], [6], [7], [8]]; however, the shortage of donors still remains [[9], [10], [11]].

Human pluripotent stem cell (hPSCs)-derived pancreatic islets are an attractive source of cell replacement therapy for diabetes. Because undifferentiated hPSCs possess the ability to proliferate infinitely, hPSCs-derived islets (hSC-islets) could potentially overcome the shortage of pancreatic islet donors. For three decades, there has been a growing body of differentiation methods for hPSCs into pancreatic beta-like cells [[12], [13], [14], [15], [16], [17], [18], [19], [20]], and it was recently reported that functional hSC-islets successfully ameliorated serum blood glucose levels in diabetic mice after transplantation [[21], [22], [23], [24]]. However, in vitro differentiated hSC-islets are still more immature than normal human islets in both function and maker expression [25,26]. Gene expression pattern and function of hSC-islets were shown to resemble native human islets after transplanting into rodents [27], suggesting that the hSC-islets maturate *in vivo* [26,27]. For clinical application, protocols generating homogeneous populations to prevent teratoma formation have also been developed using cell sorting or chemical treatment to remove off-target cells [28].

Although grafting of hSC-islets ameliorates diabetes in mice, several weeks are needed for the normalization of serum blood glucose levels after transplantation [[22], [23], [24]]. This blood glucose-normalizing process by hSC-islets is not yet fully understood. Previously, we confirmed blood glucose levels were finally maintained below 100 mg/dl in diabetic mice after hSC-islets were transplanted into their kidney capsule [23]. These levels alerted us to a glycemic set point because the human glycemic set point is around 90 mg/dl; the mouse glycemic set point is higher [29]. For example, the glycemic set point of the nude mouse is around 120 mg/dl, and the C57BL/6 mouse, around 150 mg/dl [29]. Therefore, blood glucose levels below 100 mg/dl implied that these levels were controlled by a human glycemic set point, not a mouse one. Alejandro's group showed that pancreatic islets acted as systemic glucostats and held the instructions for setting the glycemic set point [29]. We assume if transplanted hSC-islets mature functionally in mice, glycemic set point may transit from mouse to human levels. We also assume that, if both human and mouse beta cells exist together and behave independently in mice, the human beta cells will secrete more insulin to keep the blood glucose levels near the human glycemic set point**,** which is lower than mouse.

The purpose of this study is to analyze the blood glucose-normalizing process induced by grafted hSC-islets and elucidate the timing of maturation and the relation between maturation and glycemic set point. In this research, we injected STZ into mice at a different time points after hSC-islet transplantation. This approach enabled us to examine the time window for hSC-islets to compensate the function of damaged mouse beta cells. Here we report that grafted hSC-islets can control mouse blood glucose levels within 4 weeks after transplantation. Revascularization was observed as early as one week after transplantation. Furthermore, we showed that the glycemic set point is a good index for grafted-hSC-islet function *in vivo*.

## 2 Materials and methods

### 2.1 Culture of undifferentiated human induced pluripotent stem (iPS) cells

The human iPS cell line (TkDN4-M: 4M) was a kind gift from Dr. M. Ohtsu at The Institute of Medical Science, University of Tokyo. Undifferentiated 4M were maintained on mitomycin C (MMC; FUJIFILM Wako Pure Chemical Corporation, Osaka, Japan)-treated SNL feeder cells in human iPS cell medium (DMEM/Ham's F12 (FUJIFILM Wako) supplemented with 20% Knockout Serum Replacement (KSR; GIBCO BRL, Palo Alto, CA, USA), 1x MEM nonessential amino acids (FUJIFILM Wako), 0.5x penicillin streptomycin (PS; FUJIFILM Wako), 55 μM 2-melcaptoethanol (2 ME Gibco) and 7.5 μg/ml basic fibroblast growth factor (FGF2; Peprotech, Rocky Hill, NJ, USA). For passage, 4M colonies were detached with CTK solution, dissociated into single cells, and seeded onto MMC-treated SNL feeder (ECACC, Salisbury, UK) in human iPS cell medium once a week.

### 2.2 Human iPS cell culture and differentiation

Undifferentiated human iPS cells were detached with CTK solution, rinsed with D-PBS several times, and then dissociated into single cells using Accumax (Innovative Cell Technologies, San Diego, USA). Dissociated cells were seeded at a density of 1 × 10^6^ cells/ml in a spinner type reactor (Biott) containing 30 ml of mTeSR1 (Veritas) with 10 μM ROCK inhibitor (Y-27632; Cayman Chemical) at a rotation rate of 45 rpm. Spheroids formed by cell aggregation during 2 day-culture and then were cultured in hiPS medium without FGF2 for 1 day before starting differentiation.

At stage 1 (DE: definitive endoderm), spheroids were cultured for 4 days in RPMI 1640 (Wako) supplemented with 0.25% bovine serum albumin (BSA; Sigma), 0.4x penicillin and streptomycin (PS; Wako), 1 mM sodium pyruvate (Wako), 1 x NEAA, 80 ng/ml recombinant human activin A (Peprotech) and 55 μM 2-ME. Fifty ng/ml FGF2, 20 ng/ml recombinant bone morphogenetic protein 4 (BMP4; Peprotech) and 3 μM CHIR99021 (Biovision) were added for the first 2 days, and 0.5% KSR was added on Day 4 as previously reported [18].

At stage 2 (PGT: primitive gut tube), spheroids were cultured for 3 days in RPMI 1640 supplemented with 0.25% BSA, 1 mM sodium pyruvate, 1x NEAA, 0.4x PS, and 50 ng/ml recombinant human FGF7 (Peprotech), 1% B27 supplement (GIBCO) and 1:333 insulin, transferrin, selenium, ethanolamine solution (ITS-X; Gibco). The medium was changed on the third day.

At stage 3 (PFG: posterior foregut), spheroids were cultured in DMEM (8 mM glucose) supplemented with 0.15% BSA, 0.4x PS, 1x NEAA, 50 ng/ml FGF7, 1% B27 supplement, 1:333 ITS-X, 0.5 μM EC23 (Santa Cruz Biotechnology), 0.2 μM LDN 193189 (Cayman Chemical), 0.3 μM indolactam V (ILV; Cayman Chemical), and 0.25 μM SANT1 (Cayman Chemical) for 4 days. The medium was changed every 2 days during stage 3.

At stage 4 (PP: pancreatic progenitor), spheroids were cultured in DMEM (8 mM glucose) supplemented with 0.15% BSA, 0.4x PS, 1x NEAA, 50 ng/ml recombinant human FGF10 (Peprotech), 1% B27 supplement, 1:333 ITS-X, 0.2 μM EC23, 0.2 μM LDN 193189, 0.3 μM ILV, and 0.25 μM SANT1, 10 μM Alk5 inhibitor II (Rep Sox; Biovision) and 5 μM ZnSO_4_ (Sigma) for 3 days. The medium was changed on the third day.

At stage 5 (EP: endocrine progenitor), spheroids were cultured in DMEM (20 mM glucose) supplemented with 0.15% BSA, 0.4x PS, 1x NEAA, 20 ng/ml recombinant human epidermal growth factor (EGF; Peprotech), 1% B27 supplement, 1:333 ITS-X, 0.02 μM EC23, 0.2 μM LDN 193189, 0.25 μM SANT1, 10 μM Rep Sox, 5 μM ZnSO_4_, 50 ng/ml exendin-4 (Abcam), 10 μg/ml heparin (Sigma), 10 μM Y27632, 0.5 μM DBZ (Cayman Chemical) and 5 mM Nicotinamide (Sigma) for 7 days; the medium was changed every 2 days during stage 5.

At stage 6 (BETA: βcell stage), spheroids were cultured in DMEM (20 mM glucose) supplemented with 0.15% BSA, 0.4x PS, 1x NEAA, 1% B27 supplement, 1:333 ITS-X, 10 μM Rep Sox, 5 μM ZnSO_4_, 50 ng/ml exendin-4, 10 μg/ml heparin, 5 mM Nicotinamide, 10 ng/ml BMP4, 50 ng/ml recombinant human hepatocyte growth factor (HGF; Peprotech), 50 ng/ml insulin-like growth factor 1 (IGF-1; Peprotech), 1 μM R428 (Cayman Chemical) and 5 μM forskolin (Wako) for 10 days. The medium was changed every 2 days during stage 6.

**Fig. 1:**
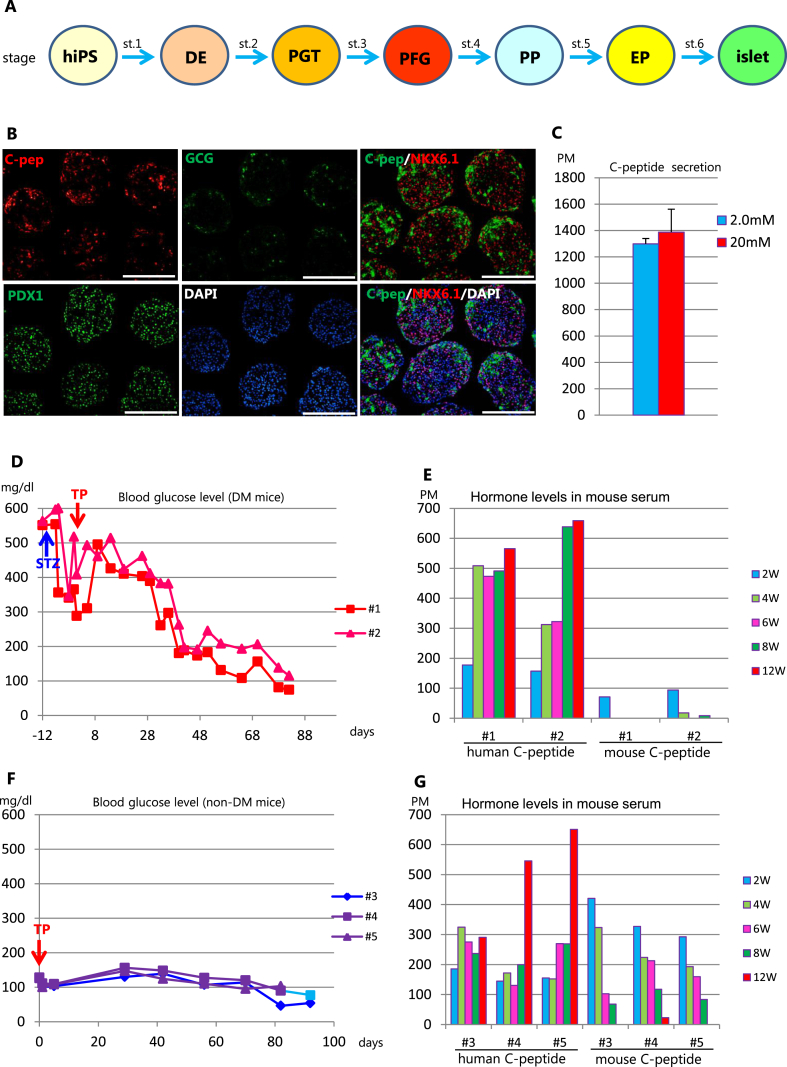
Characterization of hSC-islets before transplantation, and non-fasting blood glucose and hormone levels of DM or non-DM mice after transplantation of hSC-islets. (A) Scheme of hSC-islets differentiation by 6 step protocol. (B) Immunocytochemistry of hSC-islets before transplantation. Left upper panel: red; human C-peptide positive cells, middle upper panel: green; glucagon positive cells, left lower panel: green; PDX1 positive cells, middle lower panel: blue; nuclear staining with DAPI, right upper panel: red; NKX6.1, green; C-peptide, right lower panel: red; NKX6.1, green; C-peptide, blue; DAPI Scale bar = 200 μm (C) Glucose stimulated C-peptide secretion assay. Y-axis: The concentration of C-peptide secreted in the supernatants. We measured the concentration of C-peptide for each batch twice and calculated the mean ± SEM. (D) Periodic non-fasting blood glucose levels in diabetic mice. (E) Periodic human and mouse C-peptide levels in mouse serum. (F) Periodic non-fasting blood glucose levels in non-diabetic mice. (G) Periodic human and mouse C-peptide levels in mouse serum. TP: transplantation; STZ: streptozotocin.

**Fig. 2:**
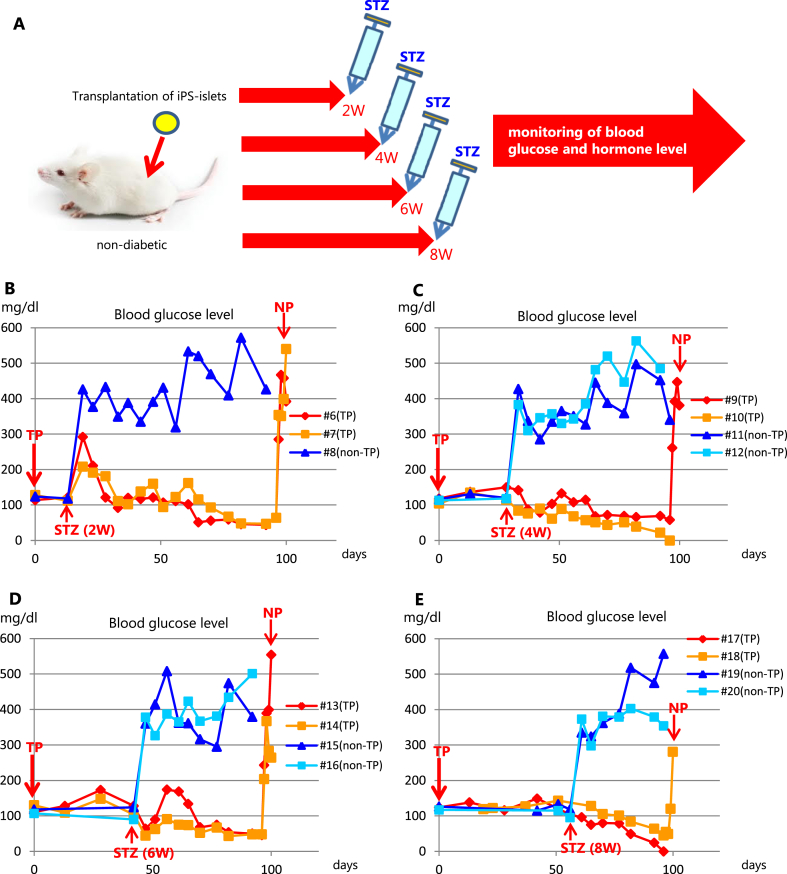
Periodic non-fasting blood glucose levels in STZ-injected mice. (A) Scheme of experimental design. STZ was administered at the indicated times after hSC-islet transplantation, and blood glucose was monitored. (B) Mice #6 and #7 were administered STZ 2 weeks after transplantation. Mouse #8 is a sham. (C) Mice #9 and #10 were administered STZ 4 weeks after transplantation. Mice #11 and #12 are shams. (D) Mice #13 and #14 were administered STZ 6 weeks after transplantation. Mice #15 and #16 are shams. (E) Mice #17 and #18 were administered STZ 8 weeks after transplantation. Mice # 19 and #20 are shams. NP: nephrectomy; TP: transplantation; STZ: streptozotocin.

**Fig. 3:**
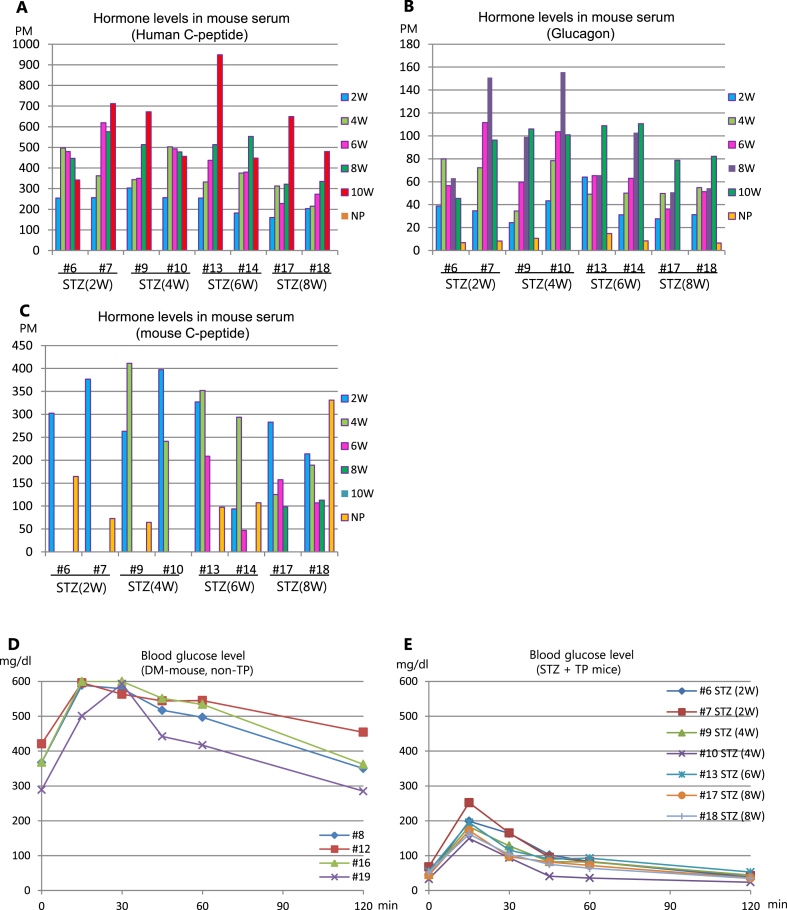
Periodical hormone levels in mouse serum and oral glucose tolerance test (OGTT). (A–C) Serum hormone levels at 2, 4, 6, 8, 10 weeks after transplantation and after nephrectomy (NP) in the same mice described in Fig.2. (A) Human C-peptide levels. (B) Glucagon levels. (C) Mouse C-peptide levels. (D–E) OGTT was performed using STZ-treated mice at 11 weeks after transplantation. Blood glucose levels were measured at 30, 60, 90, and 120 min after oral glucose administration. (D) Blood glucose levels of non-transplantation diabetic mice. (E) Blood glucose levels of hSC-islet- transplanted mice injected with STZ with different timing. OGTT: Oral glucose tolerance test.

**Fig. 4:**
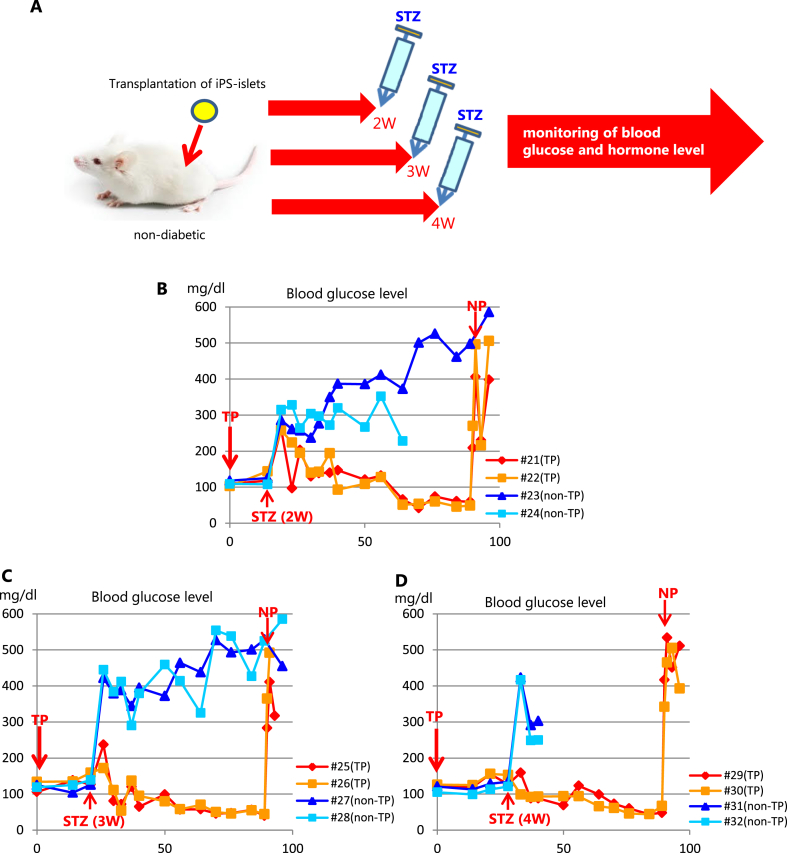
Periodical non-fasting blood glucose levels in STZ-injected mice. (A) Scheme of experimental design. STZ was administered at the indicated timing after hSC-islets transplantation, and blood glucose was monitored. (B) Mice #21 and #22 were administered STZ 2 weeks after transplantation. Mice #23 and #24 are shams. (C) Mice #25 and #26 were administered STZ 3 weeks after transplantation. Mice #27 and #28 are shams. (D) Mice #29 and #30 were administered STZ 4 weeks after transplantation. Mice #31 and #32 are shams.

**Fig. 5:**
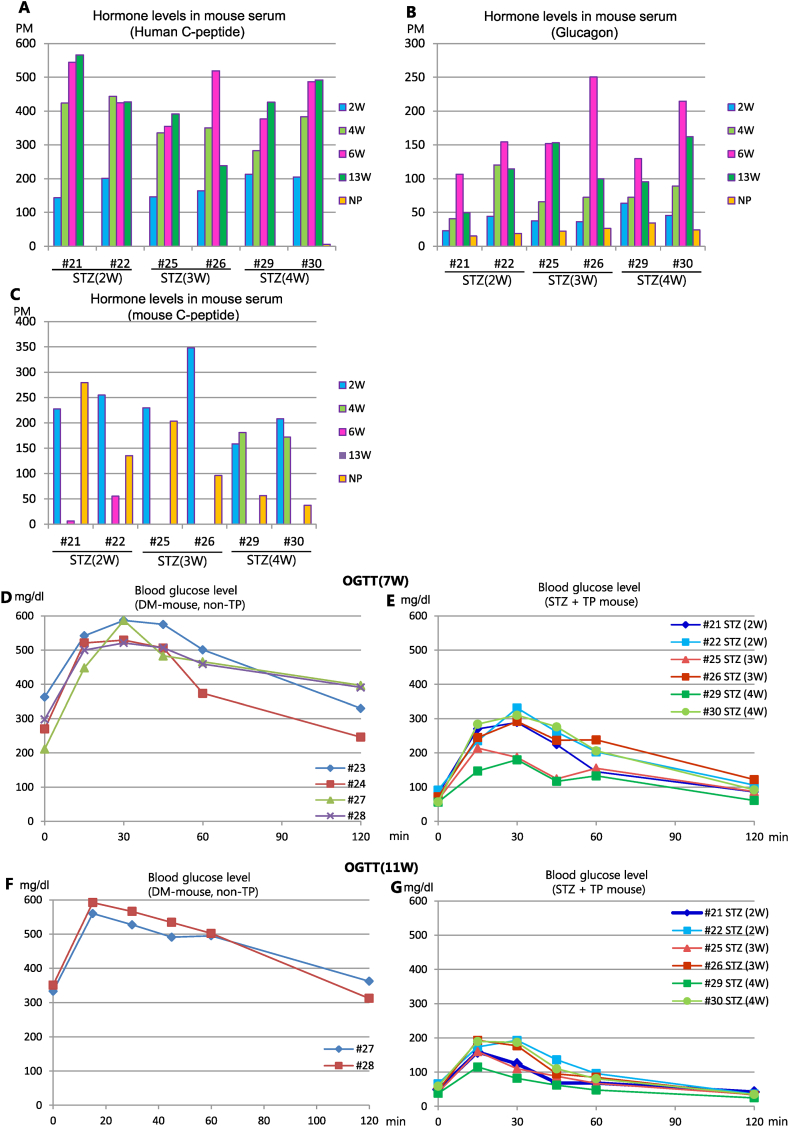
Periodical hormone levels in mouse serum and OGTT. (A–C) Serum hormone levels at 2, 4, 6, 13 weeks and after nephrectomy in the same mice described in Fig.4. (A) Human C-peptide levels. (B) Glucagon levels. (C) Mouse C-peptide levels. (D, E) OGTT was performed using STZ-treated mice at 7 weeks after transplantation. Blood glucose levels were measured as in Fig.3. (D) Blood glucose levels of non-transplantation diabetic mice. (E) Blood glucose levels of hSC-islets transplanted mice which were injected with STZ with different timing. (F, G) OGTT was performed at 11 weeks after transplantation. (F) Blood glucose levels of non-transplantation diabetic mice. (G) Blood glucose levels of hSC-islets transplanted mice which were injected STZ with different timing.

**Fig. 6:**
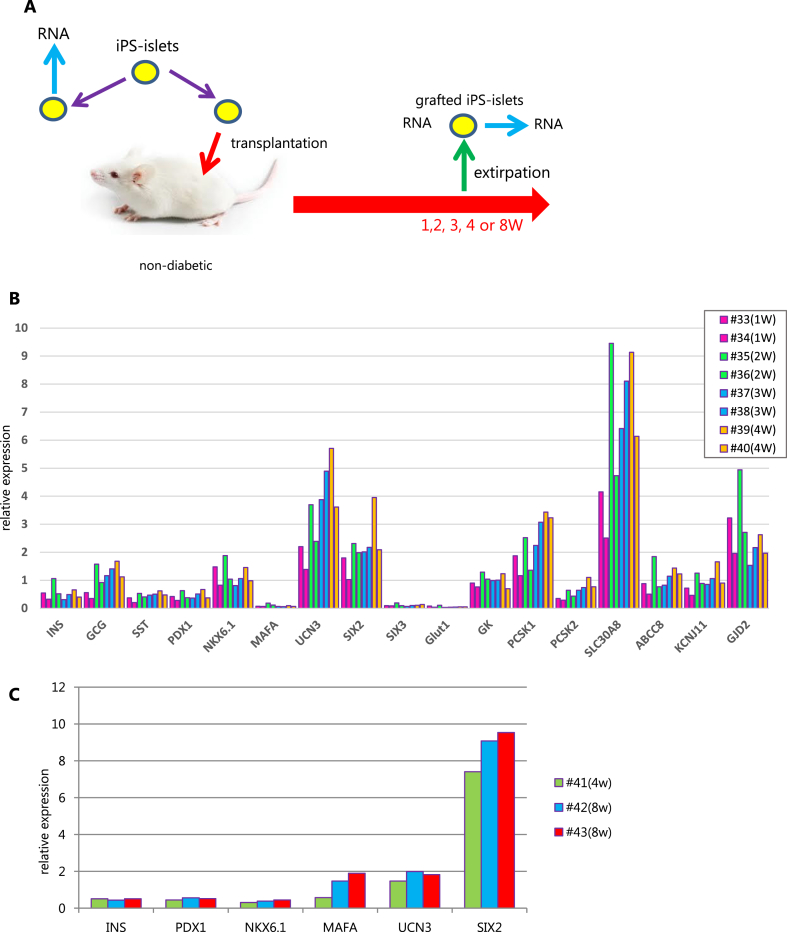
Gene expression patterns in grafted hSC-islets at 1, 2, 3, 4 or 8 weeks after transplantation. (A) Scheme of experimental design. Grafted hSC-islets were extirpated from 2 mice each at 1, 2, 3, 4, or 8 weeks, respectively. Gene expression was examined by qRT-PCR. (B) Gene expression in grafted hSC-islets at 1, 2, 3, or 4 weeks. (C) Gene expression in grafted hSC-islets at 4 or 8 weeks.

**Fig. 7:**
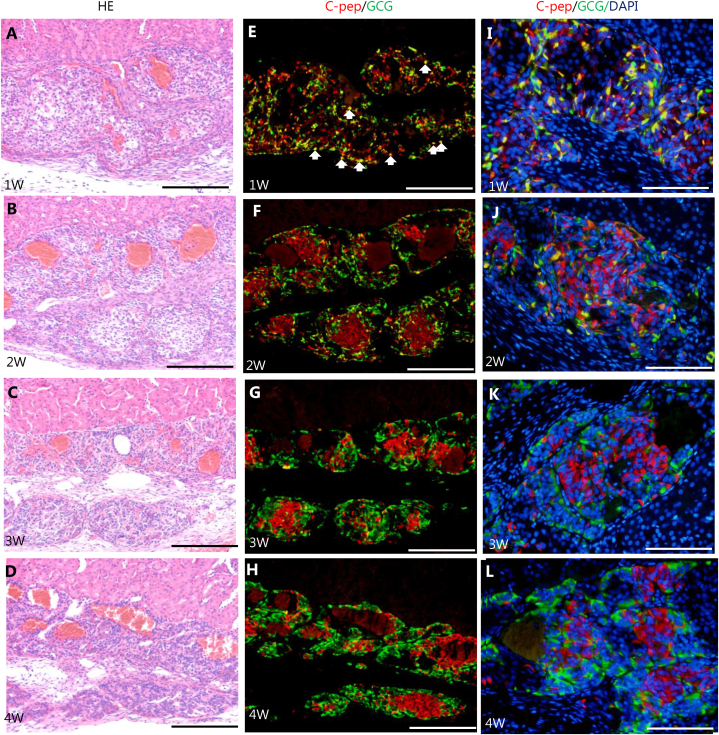
Periodical histological analysis of grafted hSC-islets. (A–D) Hematoxylin and eosin staining. (A) 1 week after transplantation. (B) 2 weeks after transplantation. (C) 3 weeks after transplantation. (D) 4 weeks after transplantation. Revascularization of grafted hSC-islets was observed from 1 week after transplantation. (E–H) Immunostainings corresponding to each H&E staining. Red indicates insulin C-peptide expression. Green shows glucagon expression. (E) 1 week after transplantation. Many insulin C-peptide and glucagon doubly positive cells (white arrows) were observed. (F) 2 weeks after transplantation. (G) 3 weeks after transplantation. (H) 4 weeks after transplantation. Scale bar = 200 μm. (I–L) High magnification image of immunostaining (I) 1 week. (J) 2 weeks. (K) 3 weeks. (L) 4 weeks. Red; C-peptide, green; GCG, blue; DAPI. Scale bar = 100 μm.

In this research, we differentiated 6 batches of hSC-islets (batch A-F). Each batch of hSC-islets was transplanted into mice as follows: Fig. 1 (batch A), Fig. 2, Fig. 3 (batch B), Fig. 3, Fig. 4 (batch C), Fig. 5 (batch D), Fig. 6 (batch E), Fig. 7 (batch F). Representative data of characterization of these hSC-islets is shown in Fig. 1 A, B.

### 2.3 Immunostaining and immunohistochemistry

Differentiated islet-like cells from human iPS cells (hSC-islets) were fixed in 4% paraformaldehyde at room temperature (RT) for 30 min. Grafted-hSC-islets were harvested from mice and were fixed in 4% paraformaldehyde at 4 °C overnight. These cells were washed with phosphate-buffered saline (PBS) and gradually dehydrated using 70% ethanol and 99.9% ethanol. Then 99.9% ethanol was replaced with xylene, and cells were embedded in paraffin and cut into 3-μm sections. These sections were mounted on slides and immersed in xylene, next in ethanol, and finally rehydrated with tap water. Hematoxylin and eosin staining was conducted according to the standard protocol. For immunostaining, sections were washed with PBS and incubated with blocking buffer (3% BSA in PBS) at RT for 1 h. Next, sections were incubated with primary antibodies (diluted in PBS containing 1.5% goat serum) overnight at 4 °C in a humidified chamber. The following primary antibodies were used: rat anti-C-peptide (1:200; DSHB, University of Iowa), rabbit anti-proglucagon (1:300; Cell Signaling Technology), goat anti-PDX1/IPF-1 (1:100; R&D), and rabbit anti-Synapsin1/2 (1:300; Synaptic Systems, Germany). Sections were washed with PBS and then incubated with a fluorescence-conjugated secondary antibody (diluted in PBS containing 1.5% goat serum) for 120 min at RT. The following secondary antibodies were used: Alexa Fluor 488-conjugated donkey anti-goat IgG (1:400; Invitrogen), Alexa 594-conjugated goat anti-rat IgG (1:400; Invitrogen) and Alexa Fluor 488-conjugated goat anti-rabbit IgG (1:400; Invitrogen). These sections were rinsed with PBS, and then phase-contrast or fluorescent images were taken with a charge-coupled device (CCD) camera (DP71; Olympus). The positive rate in Fig. 1 was calculated with Metamorph image analysis software (Molecular Devices, CA, USA), and doubly positive cells were counted by visual inspection.

### 2.4 Quantitative reverse transcription polymerase chain reaction (qRT-PCR)

Extraction and purification of total RNA were performed using RNAiso Plus (Takara, Japan). Then cDNA was synthesized with random nonamer and oligo dT(18) using PrimeScript II reverse transcriptase (Takara). qPCR was conducted using GoTaq qPCR mix and run on a CFX96 Touch Deep Well (Bio-Rad, Hercules, CA, USA). Relative quantification was done by standard curve method, and the expression levels of target genes were normalized against that of the reference gene, ornithine decarboxylase antizyme (OAZ1).

### 2.5 Animal studies

All animal experiments were approved by the Animal Care and Use Committee in the National Center for Global Health and Medicine and conducted in accordance with institutional procedures, national guidelines, and the relevant national laws on the protection of animals. Eight-week-old male NOD/SCID mice were purchased from Japan Clea and kept on a 12-h light/dark cycle with ad libitum access to drinking water and standard irradiated diet. These mice were housed for 1 week before implantation and randomly transplanted with hSC-islets. Mice were anesthetized with a mixture of medetomidine (Nippon Zenyaku Kogyo, Fukushima, Japan), midazolam (Sando, Tokyo, Japan), and butorphanol (Meiji Seika Pharma, Tokyo, Japan)**,** and 6 × 10^6^ differentiated cells were transplanted under the kidney capsule before or after STZ injection. Mice were anesthetized with inhalable isoflurane (Pfizer, NY, USA). To create diabetic model mice, mice (Japan Clea) were administered one shot of 130 mg/kg of streptozotocin (STZ; Sigma) intravenously. Transplanted kidneys were extirpated from mice around 90 days after implantation**,** and histochemical analysis was performed. All mice were anesthetized with inhalable sevoflurane (Maruishi Pharmaceutical, Osaka, Japan) and euthanized by cervical dislocation at the time of sacrifice. Blood was collected via the tail vein**.** Non-fasting blood glucose levels were examined using a glucose test kit (Glutest Neo Sensor, Sanwa Chemical). No mice reached the experimental endpoint.

### 2.6 Oral glucose tolerance test

Seven or eleven weeks after transplantation, mice were fasted for 4 h, and blood glucose was measured 15, 30, 45, 60, and 120 min after the oral administration of a glucose solution (2.0 g/kg). We also examined STZ-induced DM control NOD/SCID mice.

### 2.7 Measurement of serum hormones

Blood samples were gathered from tail vein in heparin-coated capillaries. Plasma was separated after centrifugation (10 min, 4 °C, 800 g) and kept frozen at −80 °C until measurement. Human or mouse C-peptide and glucagon concentrations in mouse plasma were determined using human ultrasensitive C-peptide ELISA kits (Mercodia), mouse C-peptide Elisa kits (Takara), and glucagon ELISA kits (Mercodia).

### 2.8 Glucose stimulated C-peptide secretion assay

Differentiated hSC-islets were pre-cultured in DMEM containing 2 mM glucose, 0.1% BSA and 10 mM HEPES at 37 °C for 1hr. These cells were then incubated in the same medium at 37 °C for 1hr, and the supernatant was harvested. Next, they were incubated in the DMEM containing 20 mM glucose, 0.1% BSA and 10 mM HEPES at 37 °C for 1hr. Then the supernatant was retrieved. After the assay, these cells were enzymatically dissociated and counted. Concentration of C-peptide in the supernatant was measured using human C-peptide ELISA kit, and the numbers of cells were counted after the assay. The concentration was adjusted per 2 × 10^6^ cells.

## 3 Results

### 3.1 hSC-islets substitute for mouse beta cells

hSC-islets were differentiated using 6 stage differentiation protocol and characterized before transplantation (Fig. 1A). We immunohistochemically confirmed that insulin C-peptide, PDX1 or GCG positive cells existed in hSC-islets (Fig. 1 B). We also observed NKX6.1 and C-peptide double positive cells (Fig. 1B, the rate of double positive cells was 18%). Next, to examine their ability to secrete C-peptide, we carried out glucose-stimulated C-peptide secretion assays. We detected a certain amount of C-peptide secretion (over 1000 pM), although this C-peptide concentration did not differ much between 2 mM and 20 mM glucose stimulation (Fig. 1C). Our previous report showed that it takes more than 6 weeks to normalize the blood glucose levels in diabetic mice after transplanting hSC-islets under the kidney capsule [23]. At that time, we noticed that their normalization was delayed even if hSC-islets produced a large amount of insulin. To investigate these phenomena, we monitored the non-fasting blood glucose and serum levels of both human and mouse C-peptide periodically after transplanting hSC-islets into STZ-treated diabetic mice (DM) or non-treated mice (non-DM). As shown in Fig. 1D, blood glucose levels of DM mice started to decrease from 4 weeks after transplantation and reached a human glycemic set point (below 100 mg/dl) 10 weeks later. Their serum human C-peptide levels increased from 150 pM 2 weeks after transplantation to 500 pM after 8 weeks, while serum mouse C-peptide levels were below 100 pM after 2 weeks and became very low thereafter, near the detection limit of ELISA (Fig. 1E). These results indicated that STZ treatment destroyed most of the mouse beta cells and mouse insulin was minimally produced. In the non-DM mice, blood glucose levels gradually decreased from 150 mg/dl to 100 mg/dl (a human glycemic set point) within 10 weeks after transplantation (Fig. 1F). Their serum human C-peptide levels tended to increase over time (Fig. 1G). Surprisingly, their serum mouse C-peptide levels continued to decrease and were barely detected after 12 weeks without STZ injection (Fig. 1G).

### 3.2 hSC-islets function well enough to control blood glucose levels 4 weeks after transplant

Next, we ran further experiments to examine the rescue potentials of human beta cells when human and mouse beta cells coexist. To destroy the mouse beta cells, we chose STZ, because human beta cells are minimally damaged by STZ administration. STZ was administered intraperitoneally 2, 4, 6 or 8 weeks after transplanting hSC-islets under kidney capsules of NOD/SCID mice (Fig. 2A).

As shown in Fig. 2B–E, blood glucose levels rapidly elevated to more than 300 mg/dl in all non-transplanted mice (non-TP). In contrast, blood glucose levels of hSC-islet transplanted mice (TP) didn't increase, except in one group in which STZ was administered 2 weeks after transplanting hSC-islets (Fig. 2B–E). Although a transient elevation of blood glucose levels was observed in those mice (Fig. 2B), their blood glucose levels fell below 100 mg/dl 10 weeks later regardless of timing of STZ administration (Fig. 2B–E). Notably, blood glucose levels immediately elevated in all mice after their transplanted kidneys were resected (nephrectomy: NT), indicating that transplanted hSC-islets controlled blood glucose levels.

### 3.3 Increase of human C-peptide and improvement of OGTT regardless of STZ-injection timing

Next, we examined the serum hormone levels of human C-peptide, glucagon, and mouse C-peptide in these mice. Serum human C-peptide levels were approximately 200 pM at 2 weeks after transplant and increased thereafter, regardless of the timing of STZ administration (Fig. 3A). We confirmed that serum human C-peptide was not detected after removal of grafted hSC-islets by nephrectomy (Fig. 3A). Serum glucagon levels rose incrementally over time, albeit with individual differences, but they also decreased after nephrectomy, similarly to human C-peptide (Fig. 3B). Serum mouse C-peptide levels decreased in inverse proportion to human C-peptide levels after transplant in cases #17 and #18 (Fig. 3C). Although serum mouse C-peptide levels of most mice were undetectable after STZ administration, they increased once again after nephrectomy (Fig. 3C), indicating that functional mouse beta cells were present.

To further investigate the function of grafted hSC-islets *in vivo*, we performed oral glucose tolerance tests (OGTT) 11 weeks after transplantation. Non-transplantation DM mice maintained high blood glucose levels until 60 min after glucose administration (Fig. 3D). In contrast, blood glucose levels peaked below 200 mg/dl at 15 min in almost all transplanted mice and then rapidly returned to their pre-glucose challenge levels irrespective of the timing of STZ administration (Fig. 3E). These data suggest that the grafted hSC-islets functioned robustly *in vivo.* We did not observe any differences in their function caused by the timing of STZ injection.

### 3.4 Four weeks are necessary for hSC-islets to function

We found that the function of grafted hSC-islets improved between 2 and 4 weeks after implantation. To narrow the time window between 2 and 4 weeks, we injected STZ into mice at 2, 3, or 4 weeks after grafting (Fig. 4A). Blood glucose levels of the non-grafted mice rapidly elevated with the STZ treatment and then maintained high levels (Fig. 4B–D); two of these mice died around 40 days(Fig. 4D). In contrast, when STZ was injected into mice at 2 weeks after grafting, their blood glucose increased transiently to between 200 and 300 mg/dl but then decreased below the human glycemic set point (Fig. 4B). When STZ was injected into mice 3 weeks after transplantation, their blood glucose levels increased to around 200 mg/dl; these levels were lower than that the mice administrated STZ 2 weeks after transplantation. Of note, these mice reached the human glycemic set point earlier than those administered STZ 2 weeks after transplantation (Fig. 4C). We observed again that serum blood glucose levels were seldom affected by the administration of STZ 4 weeks after transplantation and then similarly reached the human glycemic set point (Fig. 4D). Blood glucose levels in all transplanted mice rose rapidly to diabetic levels after nephrectomy. These results indicate that it takes at least 4 weeks for grafted hSC-islets to control the serum blood glucose levels and more time to reach the human glycemic set point.

### 3.5 The function of hSC-islets improves until 11 weeks after transplantation

Again, we measured human or mouse C-peptide and glucagon levels in mouse serum. Human C-peptide levels were approximately 150–200 pM 2 weeks after grafting and then increased above 300 pM 4 weeks later (Fig. 5A). After 6 weeks, human C-peptide levels peaked or reached a constant level, depending on the individual mouse (Fig. 5A). Serum glucagon levels peaked at 6 weeks and then downregulated at 13 weeks. Moreover, reduced serum glucagon levels were observed after nephrectomy (Fig. 5B). Although serum mouse C-peptide levels were over 150 pM at 2 weeks just before STZ injection, STZ injection strongly reduced these levels (Fig. 5C). However, the mouse C-peptide levels increased again after nephrectomy (Fig. 5C). To further test the function of grafted-hSC-islets *in vivo*, we conducted OGTT at 7 and 11 weeks after grafting (Fig. 5D–G). In non-transplanted DM mice, serum blood glucose levels remained over 450 mg/dl for 60 min and then did not decrease below 300 mg/dl after glucose load (Fig. 5D, F). In contrast, the peak of serum blood glucose levels did not exceed 300 mg/dl during the first 30 min**,** and blood glucose levels returned to 100 mg/dl 120 min after glucose challenge in transplanted mice when OGTT was performed 7 weeks after implantation (Fig. 5E). Furthermore, when OGTT was performed 11 weeks after grafting, the peak of serum blood glucose levels did not exceed 200 mg/dl during first 30 min, and serum blood glucose levels fell below 100 mg/dl 60 min after glucose challenge (Fig. 5G). These data suggest that grafted-hSC-islets functioned *in vivo* irrespective of the timing of STZ administration, and that their function further improved from 7 to 11 weeks.

### 3.6 Gene expression of maturation markers in grafted-hSC-islets

Because the results of OGTT clearly demonstrated that grafted-hSC-islets maturated *in vivo*, we focused on the surrounding environment at the grafted site. We extirpated grafts at 1, 2, 3, 4, or 8 weeks after transplantation and tested the gene expression patterns of grafted hiPS-islets by RT-qPCR (Fig. 6A). We examined the maturation and functional markers for beta cells such as UCN3, SIX2, PCSK1, PCSK2, SLC30A8, ABCC8 and KCNJ11 during the first 4 weeks (Fig. 6B). Maturation markers MAFA, UCN3 and SIX2 tended to increase from 4 to 8 weeks (Fig. 6C).

### 3.7 Revascularization occurred within 1 week for grafted hSC-islets

We periodically examined the revascularization of grafted hSC-islets during the first 4 weeks after grafting (Fig. 7A–D). Capillary vessels were already present in the grafted area 1 week after transplantation, and many dilated capillaries had also appeared at 2, 3, and 4 weeks. We also noted that vascularization did not occur at areas distant from the transplantation site in the kidney capsule in the mouse even 2 weeks after transplantation (data not shown). To further analyze the state of pancreatic beta and alpha cells in grafted hSC-islets during this period, we utilized immunohistochemistry using C-peptide and glucagon antibodies (Fig. 7E-L). Notably, there were many C-peptide and glucagon double-positive cells as well as C-peptide single positive cells at 1 week (Fig. 7E, arrows). There were 66 doubly positive cells in this section (Fig. 7I). From 2 weeks onward, C-peptide and glucagon doubly positive cells decreased (24 cells as shown in Fig. 7J); in contrast, C-peptide or glucagon singly positive cells increased (Fig. 7F-H, J-L). We could not find any doubly positive cells by 3 weeks after transplantation (Fig. 7K. L).

### 3.8 No reinnervation was detected in grafted hSC-islets

**Fig. 8:**
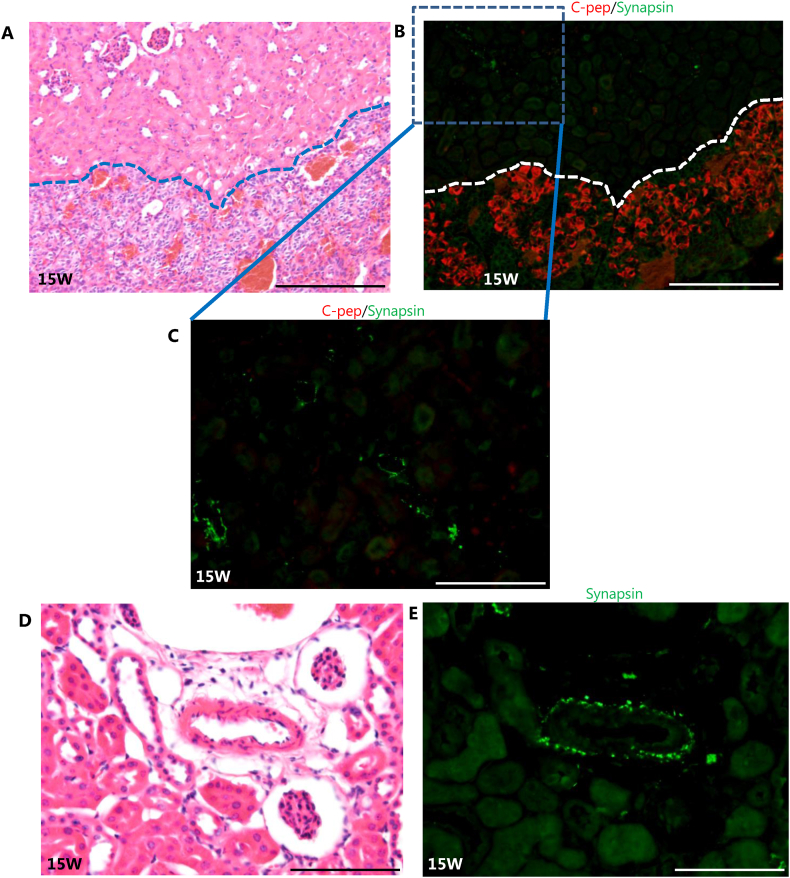
Reinnervation around the grafted area in the mouse kidney at 15 weeks after transplantation. (A) Hematoxylin and eosin staining. Renal parenchyma is shown above dotted lines, and grafted hSC-islets are shown below them. (B, C) Immunostaining corresponding to (A). Red indicates insulin C-peptide expression. Green shows synapsin expression. (C) High magnification image of rectangular area surrounded by dotted line in (B). (D) Hematoxylin and eosin staining of the renal parenchyma. (E) Immunostaining corresponding to (D). Synapsin expression was detected around the blood vessels in the renal parenchyma. Scale bar = 200 μm (A, B), 100 μm (C, D, E).

The pancreatic islet is innervated by an autonomic axon, and neural input influences its function [[30], [31], [32]]. Innervation of grafted mouse islets was initiated by 6 weeks and then increased over time [33], suggesting that the function of grafted-hiPS-islets responds to reinnervation. Using the neural marker synapsin, we examined whether innervation occurred in grafted-hSC islets by immunohistochemistry (Fig. 8). We observed many C-peptide positive beta cells in grafted hSC-islets, but we could not detect any synapsin-positive neural fibers near beta cells (Fig. 8A, B, C). However, synapsin positive cells were detected in renal parenchyma or around blood vessels in renal parenchyma, indicating that this antibody worked properly (Fig. 8D and E). These data suggest that innervation of grafted hSC-islets did not contribute to the enhancement of their function until 15 weeks.

## 4 Discussion

In this study, we examined the blood glucose-regulating function of grafted hSC-islets using a diabetic and non-diabetic mouse model by changing the timing of STZ injection. We also focused on the glycemic set points of both human and mouse and the relationship between blood glucose levels and insulin secretion from grafted hSC-islets. We demonstrated that grafted hSC-islets were able to control STZ-mediated hyperglycemia within 4 weeks after transplantation and finally reached the human glycemic set point within 10 weeks. We confirmed the elevation of maturation markers of beta cells in mRNA levels after 4 weeks and the improved function of beta cells over time by OGTT. Although revascularization was observed as early as one week after transplantation, no reinnervation was observed in the grafted hSC-islets even after 15 weeks.

In the present study, we adopted two kinds of streptozotocin (STZ)-induced diabetes mice models to examine the time course of maturation and the effect of hyperglycemia on the maturation. In the pre-existing diabetes model, hyperglycemia is induced by STZ injection before transplantation. In the other model, hSC-islets were implanted in non-diabetic mice in advance, and STZ was administered later. This latter model utilizes the specific function of STZ that selectively destroys mouse pancreatic beta cells. Grafted hSC-islets suffer minimal damage because human beta cells barely express Glut2, which transports STZ into pancreatic beta cells [[34], [35], [36], [37]]. This advantage allows us to choose the timing of STZ administration for examining the time course of maturation.

Although there are some reports in which STZ was injected after cell transplantation [12,38], to our knowledge, this is the first study that examines the timing of STZ administration after cell transplantation (1–2 week intervals). Once diabetes was provoked by STZ administration, it took more than 10 weeks to normalize the blood glucose level after hSC-islet transplantation (Fig. 1). However, the grafted hSC-islets prevented STZ-mediated hyperglycemia within 4 weeks after transplantation, even when the standard dosage of STZ for inducing diabetes was administered (Fig. 2, Fig. 4). This time point, “4 weeks”, corresponded to the descending timing of blood glucose level when hSC-islets were transplanted into diabetic mice. These results indicate that the grafted-hSC islets had acquired the ability to adjust STZ-induced hyperglycemic activity by secreting enough insulin. Even though the transplanted hSC-islets suppressed the elevation of blood glucose after STZ injection, it took several more weeks to stabilize the blood glucose levels below 100 mg/dl, which we regard as the human glycemic set point. In other words, the hSC-islets had fully matured and functioned like native human islets at this point.

When hSC-islets were transplanted in non-DM mice, serum human C-peptide levels increased over time, while serum mouse C-peptide levels decreased and were barely detectable at 12 weeks, as shown in Fig. 1G. These results suggest that mouse beta cells stop producing insulin without receiving any damage when human beta cells are alternatively producing sufficient insulin. Grafted-hSC islets continued to secrete insulin to reach the human glycemic set point, but native mice didn't need to secrete insulin below the mouse glycemic set point, which is higher than human. Our data shown in Fig. 3C and 5C as well as Fig. 1G clearly demonstrate that this is the case, and another study also supports this observation [29]. In contrast, when hSC-islets were transplanted into diabetic mice, it took longer to reach the human glycemic set point than those transplanted into non-diabetic mice. One possible reason is that the glucotoxity of the hyperglycemia may have caused some damage to the grafted hSC-islets. Judging from the human C-peptide levels in mouse serum 2 weeks after transplantation, glucotoxicity may not be the main cause at the initial stage, if at all, because human C-peptide levels were almost the same in both DM mice and non-DM mice. Another possible factor is the mode of insulin secretion**,** because pulsatile secretion is reported to enhance insulin action and effectively regulate blood glucose levels better than constant insulin secretion [39,40]. Also, hub/leader cells are suggested to be necessary to communicate and orchestrate mutual beta cells for pulsatile insulin secretion [[41], [42], [43]]. Hub/leader cells might be damaged by pro-inflammatory cytokines associated with diabetes, because hub/leader cells are targeted by a diabetic milieu [41]. Generation of hub/leader cells in grafted-hSC-islets may be also affected by diabetic state. Further research is needed for better understanding of hub/leader cells.

In terms of maturation of grafted hSC-islets, environmental factors in the tissue surrounding grafted sites, such as revascularization and reinnervation, are thought to be important. A pancreatic islet is highly vascularized, and paracrine signals and extracellular matrix (ECM) stemming from a vessel are important for pancreatic beta cell function [[44], [45], [46], [47]]. Although revascularization in grafted-hSC islets or endocrine progenitors has been observed in some studies [22,23,48], chronological analysis of revascularization in grafted-hSC islets has not been examined. Therefore, it is important to show revascularization occurred in grafted-hSC islets as in native islets, in which revascularization is initiated within 1 week after transplantation [[49], [50], [51], [52], [53], [54]].In fact, we found revascularization in subrenal capsules of mice as early as 1 week after grafting, which is consistent with previous reports [[49], [50], [51], [52], [53], [54]]. Interestingly, maturation of grafted hSC-islets at the cellular level was observed by chronological examination, because immunohistochemical analyses revealed the transition from polyhormonal cells to monohormonal cells, as shown in Fig. 7. Moreover, the trend of increasing gene expression levels of maturation markers of beta cells suggested the maturation process during first 4 weeks. Our qPCR data also suggested the upregulation of genes related to insulin granule processing/maturation, such as PCSK1 and SLC30A8. Therefore, improvement of grafted hSC-islet function may be accompanied by insulin granule maturation.

As for innervation, mouse pancreatic endocrine cells are innervated by autonomic nerves *in vivo* [[30], [31], [32]], and C57BL/6 mouse pancreatic islets are influenced directly by parasympathetic nerve innervation [55]. Anatomical studies indicate that human pancreatic endocrine cells are also innervated by autonomic nerves [30]. However, human pancreatic endocrine cells are not highly innervated by autonomic axons; this is a marked contrast to mouse pancreatic endocrine cells [30]. When mouse pancreatic islets were transplanted into mice, re-innervation was reported 6 weeks after grafting [33], but, to our knowledge, re-innervation of grafted hSC-islets has not yet been examined. Since reinnervation occurred later than revascularization, we searched for reinnervation until 15 weeks after grafting. However, we did not detect any innervation around the grafted hSC islets. This might reflect the innervation pattern of human islets; native human islets grafted into eyes were not innervated [55]. Furthermore, the blood vessels in the grafted hSC-islets area were not invaded by nerve fiber, presumably because these blood vessels originated from the mouse. Taken together, we conclude that the function of grafted hSC-islets was not affected by autonomic nerves, unlike the situation in the normal mouse.

The limitation of this study was the small sample size for each group in the animal experiments. We used two mice for each group, and we presented individual mouse data, which were consistent. Indeed, we performed experiments twice for STZ injection 2 and 4 weeks after transplantation and obtained reproducible results. Although the limited sample size precludes us from performing statistical analysis, we observed that each mouse's value had the same trend in each group. Despite this limitation, our findings have important implications for hSC-islets transplantation therapy. Confirming whether grafted-hSC–islets can set blood glucose to the human glycemic set point in mice provides a better evaluation of the function of the hSC-islets, differentiation protocols, and hPSC line used for hSC-islets. Although reduction in blood glucose levels by hSC-islets transplantation in diabetic mice has been reported, studies to ameliorate blood glucose levels to the human glycemic set point are not so many. Controlling blood glucose levels to the human glycemic set point reflects full maturity of the grafted-hSC islets. Moreover, we found that blood glucose levels reached the human glycemic set point by grafted-hSC islets regardless of innervation. This observation implies that transplantation sites or methods such as naked or device are selectable without consideration for reinnervation after transplantation.

In summary, the human glycemic set point are a good index for the maturity of grafted hSC-islets *in vivo*. Further research is needed to fully elucidate the maturation process and the relationships between the glycemic set point and grafted hSC functions. We think that not only single cell level but also mass level studies are essential for understanding maturation of grafted hSC-islets.

## Author contributions statement

Shigeharu G. Yabe: Conceived and designed experiment; Performed the experiments; Analyzed and interpreted the date; Wrote the paper.

Satsuki Fukuda, Junko Nishida, Fujie Takeda: Performed the experiments.

Hitoshi Okochi: Conceived and designed experiment; Analyzed and interpreted the date; Wrote the paper.

## Ethical approval statement

This study was approved by our institutional review board.

## Human and animal rights statement

This article does not contain any studies with human subjects.

## Research ethics and informed consent statement

All animal studies were approved by the Animal Care and Use Committee in the National Center for Global Health and Medicine (approval number:21,020). There are no human subjects in this article, so informed consent is not applicable.

## Funding statement

This work was supported by The Grant of 10.13039/100012319National Center for Global Health and Medicine (21A1017) to HO.

## Declaration of competing interest

The authors declare that they have no known competing financial interests or personal relationships that could have appeared to influence the work reported in this paper.
